# Supplementary material for: Is half the world’s population really below ‘replacement-rate’?
Source: PLoS One. 2019 Dec 2;14(12):e0224985. doi: 10.1371/journal.pone.0224985 (PMC6886770; doi:10.1371/journal.pone.0224985)
Supplement: Attachment — Submitted filename: Review for PONE-D-19-14424.pdf [file pone.0224985.s001.pdf]

Referee report for "Is half the world's population really below 'replacement-rate'? "(PONE-D-19-14424)

This paper deals with an interesting topic, i.e. evaluating the variability in contemporary replacement level of fertility instead of empirical estimated level. The paper justified the findings through illustration from different regions. The only flaw of the article is that it is too brief in some places. The following comments are my recommendations about some specific points.

1. The paper requires a careful revision for small typos.
2. TFR=2.1 is usually considered as replacement level of fertility in conventional setting (also in this paper). This value should be denoted using a consistent term all over the paper (say BRF, or some new term).
3. Please summarize the contents and explanations of the graph of Wilson in one or two sentences.
4. In addition to the three sketches, please include few more lines mentioning the importance of a variable replacement level of fertility (say, for national level policy implication).
5. In materials and methods, interpretation of NRR, $f(x)$ and $L(x)$ are missing.
6. Please express all the demographic indicators like TFR, RRF, ... in plain texts (instead of italic) all over the paper.
7. There should be two distinct part in the Materials and method section. The first one will be for methods only and the second one will be for data sources with justification notes (where necessary). The word 'exercise'should be replaced by illustration or analysis.
8. The statement "This means that 54,364,244 are, effectively, unaccounted for."is not clear.
9. In the result section, the observed TFR should be denoted with a distinct, different notation.
10. The result analysis should be stronger and it could benefit from few more graphical representation (please see the next comments). For instance, the difference between the observed TFR and RRF could be presented from other different point of views. Figure 1 is difficult to understand or compare. A plot with higher resolution is required for that.
11. The statement "Taken together, it is reasonable to assume that the majority of the world's population ... probably also TFR<2.1."is not clear. Please rephrase with further explanation. Beside, this statement is too generalizing without mentioning clear assumption or citing reference.
12. The first part of the result needs more explanation for the observed regional pattern. This global illustration is more convincing for the title of the paper than Indian or Chinese context alone. Both for the global and Indian data, a plot showing the seasonal difference (for selected years) between contemporary replacement level of fertility and RRF will be useful.
13. From the formula of RRF, clearly the variability in replacement level of fertility are attributable to its components (SR_b, $L(x)$ or ASFR). How the different components are affecting the regional scenario? I think including these component-wise detailed findings is out of scope of the current manuscript but some discussion on this should be included.
14. The discussion section will be better with some guidelines regarding further scope of research from the obtained results.
