# Supplementary material for: Is half the world’s population really below ‘replacement-rate’?
Source: PLoS One. 2019 Dec 2;14(12):e0224985. doi: 10.1371/journal.pone.0224985 (PMC6886770; doi:10.1371/journal.pone.0224985)
Supplement: Attachment — Submitted filename: Response to comments.docx [file pone.0224985.s002.docx]

Reviewer #1: This paper deals with an interesting topic, i.e. evaluating the variability in contemporary replacement level of fertility instead of empirical estimated level. The paper justified the findings through illustration from different regions. The only flaw of the article is that it is too brief in some places. The following comments are my recommendations about some specific points.

1. The paper requires a careful revision for small typos.

**Sorry for this. Done.**

2. TFR=2.1 is usually considered as replacement level of fertility in conventional setting (also in this paper). This value should be denoted using a consistent term all over the paper (say BRF, or some new term).

**Thanks. We prefer to stick with TFR=2.1 precisely because this is the concept that we are fighting against. Though, we remove BRF as an abbreviation and in our rewrite and tidying up have sought to make the flow and understanding easier.**

3. Please summarize the contents and explanations of the graph of Wilson in one or two sentences.

**Actually this discussion we have makes it more complex than it needs to be. We have rewritten it to be clearer.**

4. In addition to the three sketches, please include few more lines mentioning the importance of a variable replacement level of fertility (say, for national level policy implication).

**WE elaborate on this a little more in the discussion (albeit to say about replacement fertility may not be so important.**

5. In materials and methods, interpretation of NRR, f(x) and L(x) are missing.

**Sorry -rewritten and explained; fx is a typo and should be Fx**

6. Please express all the demographic indicators like TFR, RRF,... in plain texts (instead of italic) all over the paper.

**Done**

7. There should be two distinct part in the Materials and method section. The first one will be for methods only and the second one will be for data sources with justification notes (where necessary).

**Done**

The word "exercise" should be replaced by illustration or analysis.

**Done throughout (inlcuding in the results section) - we now use the word ‘illustration’**

8. The statement "This means that 54,364,244 are, effectively, unaccounted for." is not clear.

**Rewritten for greater clarity.**

9. In the result section, the observed TFR should be denoted with a distinct, different notation.

**Rather than insert new unfamilar notation, we simply refer to it as ‘observed TFR’.**

10. The result analysis should be stronger and it could benefit from few more graphical representation (please see the next comments). For instance, the difference between the observed TFR and RRF could be presented from other different point of views. Figure 1 is difficult to understand or compare. A plot with higher resolution is required for that.

**We have adapted the graphical representations and presented the data for India in Tabular form to more clearly represent the extent to which the country straddles the ‘replacement boundary’ (however defined). Higher resolution images provided.**

11. The statement "Taken together, it is reasonable to assume that the majority of the world's population ... probably also TFR<2.1." is not clear. Please rephrase with further explanation. Beside, this statement is too generalizing without mentioning clear assumption or citing reference.

**We have now actually made these calculations and present them in our rewritten Discussion section. Properly referenced and all assumptions spelt out etc.**

12. The first part of the result needs more explanation for the observed regional pattern. This global illustration is more convincing for the title of the paper than Indian or Chinese context alone. Both for the global and Indian data, a plot showing the seasonal difference (for selected years) between contemporary replacement level of fertility and RRF will be useful.

**We have almost completely rewritten the results and discussion sections to take account of precisely these remarks. We believe it is much clearer.**

13. From the formula of RRF, clearly the variability in replacement level of fertility are attributable to its components (SR_b, L(x) or ASFR). How the different components are affecting the regional scenario? I think including these component-wise detailed findings is out of scope of the current manuscript but some discussion on this should be included.

**This is rather beyond our scope here. However, we have written a much-expanded limitations section which covers issues of measurement etc.**

14. The discussion section will be better with some guidelines regarding further scope of research from the obtained results.

**We actually insert a new last line to the study which really tries to reassert our point in this paper: While most studies call for further research in a given area, we simply argue the research that is done is done correctly, and that any shortcuts we make should be very clearly defined and better justified.**

Reviewer #2: The is an interesting paper. I agree with the arguments made by the author. However, I have some minor comments which the author may consider it before the publication.

1. I am unable to get the answer in the conclusion about the title you started with. Is half world population really below replacement level? The analysis and the conclusion seems to be brief and unable to get the correct view of the authors based on the revised calculations. Though the authors have contested the other publications and methods those authors have used for their conclusions in the introduction, I am unable to get answer for it. The figure 1 is not clear to read, proportion of world population below the replacement level over time based on the revised method. It would be good, if you provide a table comparing your proportion of pop with previous authors calculations to prove that how your method has changed the previous authors conclusions in a comparative view. In the case of India, it is clearly shows that proportion below RRF is greater than proportion of pop below the TFR of 2.1. But this was not the case with world population. It is very unclear.

**Thank you very much indeed for these very clear and important suggestions. In order to account for your ideas, we have effectively rewritten the results and discussion sections to make the ‘answer’ much clearer; we have changed and adapted the presentation of the figures, and put the Indian data in a table with the RRFs. We cannot readily compare to Wilson as we are considering a different time period. We explicitly state that there is no real material difference between our findings and the examples we cite (see Discussion section); but then focus on the different methods.**

2. Though I agree with the revised method of RRF to be used for the replacement level fertility not TFR 2.1. But I have a concern, how RRF is affected by the tempo effect for a given period. We know that TFR is distorted by changes in timing of births, duration of marriage, parity distribution and at entry into sexual union. How the RRF is affected by these factors because the RRF is estimated from the period ASFR and TFR of a given year. Suppose we use the tempo adjusted TFR for computing the RRF, what would be effect on the proportion of world population below the replacement level (adjusted RRF). In other words, how it would affect your conclusions? What would be your response on this?

**Thanks very much. Very good point. We discuss this in a new limitations section.**
